# In Vitro Plant Regeneration in Conifers: The Role of *WOX* and *KNOX* Gene Families

**DOI:** 10.3390/genes12030438

**Published:** 2021-03-19

**Authors:** Natalia Bueno, Candela Cuesta, María Luz Centeno, Ricardo J. Ordás, José M. Alvarez

**Affiliations:** 1 Plant Physiology, Biotechnology Institute of Asturias (IUBA), Department of Organisms and Systems Biology, University of Oviedo, ES-33071 Oviedo, Spain; nbuenofernandez@gmail.com (N.B.); cuestacandela@uniovi.es (C.C.); rordas@uniovi.es (R.J.O.); 2 Plant Physiology, Department of Engineering and Agricultural Sciences, University of León, ES-24071 León, Spain; mlcenm@unileon.es

**Keywords:** conifers, homeobox genes, de novo organogenesis, *KNOX* genes, micropropagation, *Pinus* spp., *Picea* spp., somatic embryogenesis, *WOX* genes

## Abstract

Conifers are a group of woody plants with an enormous economic and ecological importance. Breeding programs are necessary to select superior varieties for planting, but they have many limitations due to the biological characteristics of conifers. Somatic embryogenesis (SE) and de novo organogenesis (DNO) from in vitro cultured tissues are two ways of plant mass propagation that help to overcome this problem. Although both processes are difficult to achieve in conifers, they offer advantages like a great efficiency, the possibilities to cryopreserve the embryogenic lines, and the ability of multiplying adult trees (the main bottleneck in conifer cloning) through DNO. Moreover, SE and DNO represent appropriate experimental systems to study the molecular bases of developmental processes in conifers such as embryogenesis and shoot apical meristem (SAM) establishment. Some of the key genes regulating these processes belong to the *WOX* and *KNOX* homeobox gene families, whose function has been widely described in *Arabidopsis thaliana*. The sequences and roles of these genes in conifers are similar to those found in angiosperms, but some particularities exist, like the presence of *WOXX*, a gene that putatively participates in the establishment of SAM in somatic embryos and plantlets of *Pinus pinaster*.

## 1. Introduction

Conifers constitute the largest and more diverse group of extant gymnosperms and are distributed worldwide in a great variety of ecosystems, especially in the boreal and temperate forests from North America and Eurasia, showing a great capacity to adapt to variable environmental conditions (for a complete review see [1]). Coniferous forests, which cover vast areas in the Northern hemisphere, constitute one of the largest terrestrial carbon sinks and play an important role in climate change mitigation. Conifers also have an enormous economic importance, as they are a renewable source of timber, paper pulp and other non-wood products like resins, natural oils and products with medical use (for example, the anti-cancer drug Taxol). It is estimated that 50% of the global timber is supplied by conifers, mainly by the genus *Pinus*, as they generate higher and faster economic yield than angiosperms [2]. Some conifers also are used in horticulture for its edible seeds or with ornamental purposes.

Due to the increasing wood demand, conifers have been extensively used for reforestation, and native forests have been replaced by conifer plantations in many areas of the world [2]. Human activities can disrupt forest ecosystems with the subsequent loss of the genetic diversity, which is essential for the adaptation capability to variable environmental conditions. In the climate change scenario, it must be also taken into account that natural disturbance agents are expected to have a greater impact on forests in the near future, which will be especially pronounced in coniferous forests and boreal biomes compared to broadleaved and mixed forests [3]. In particular, studies suggest that global warming is likely to increase the impact of fire, pests and pathogens on forests at a global scale, and drought will be especially severe in those areas with restricted water availability. Thus, sustainable forest management requires the development of strategies for the preservation of natural forests and the establishment of high-yield plantations with enhanced biomass production. For that purpose, breeding programs for the selection and multiplication of superior varieties with improved production traits such as growth rate, wood quality and tolerance to biotic and abiotic stresses have been implemented.

In this context, the development of effective methods for mass clonal propagation of selected genotypes acquires great importance. However, this is not achievable through techniques like grafting or coppicing in conifers [4]. Currently, micropropagation techniques, together with rooting of cuttings, are considered the most effective tools for the propagation of coniferous elite varieties at a large scale [4]. Micropropagation consists of the multiplication of plants using in vitro tissue culture, that is, through the culture of cells, tissues or organs in artificial media, usually supplemented with plant growth regulators (PGRs), under aseptic and very controlled conditions. It exploits the characteristic developmental plasticity of plants to adapt to variable environmental conditions, in particular their high regeneration capacity. Thus, under the appropriate conditions, cultured explants undergo morphogenesis and give rise to somatic embryos, through a process known as somatic embryogenesis (SE), or to adventitious shoots which are late rooted (de novo organogenesis, DNO). In both cases, either SE or DNO, the result is the regeneration of complete plants once the embryos germinate and/or the plants are acclimatized.

Domestication of coniferous species through traditional plant breeding is technically more difficult and time-consuming than other crops due to their big size, long generation times, and the prolonged juvenile stage, as most traits that are important for production only can be evaluated during the adult phase. Thus, the application of genetic engineering techniques allows to shorten the breeding process substantially. In this context, SE and DNO are essential because they make possible the regeneration of transgenic plants from explants genetically transformed with genes of interest through biolistic techniques or mediated by *Agrobacterium tumefaciens* (currently called *Rhizobium radiobacter*) (reviewed in [5]). Recently, genome editing technologies like Clustered Regularly Interspaced Short Palindromic Repeats/Cas9 have been successfully applied in several herbaceous and woody angiosperms, although no application in gymnosperms has been reported to date [6]. Apart from their use for clonal propagation and plant breeding, SE and DNO have been proven to be useful tools for basic research of developmental processes in conifers. In particular, SE in *Picea abies* has been proposed as an ideal experimental system for the study of embryo development [7]. Similarly, adventitious caulogenesis from *Pinus pinea* cotyledons has been used for the analysis of the underlying mechanisms of shoot apical meristem (SAM) establishment, as it is very repetitive, synchronous and the big size of cotyledons facilitates their manipulation [8].

The use of SE and DNO for all the mentioned purposes requires a deep understanding of their molecular basis, but molecular studies about the biology of conifers are much more difficult than in other plant lineages like angiosperms. These organisms are characterized by extraordinarily large genomes with high heterozygosity levels and high repetitive DNA content. Unlike model plant species such as *Arabidopsis thaliana*, identification of genes involved in SE and DNO in conifers through forward and reverse genetics is extremely challenging, due to the lack of defective mutants, difficulties for applying techniques like T-DNA insertional mutagenesis, and the fact that the first annotated reference genome was not available until 2013 [9]. The development of next-generation DNA sequencing technologies and powerful bioinformatics methods for the assembly and annotation of the resulting sequences allowed the obtaining of the full genome and/or transcriptome from several coniferous species (for a complete review see [10]), which has facilitated the identification of genes putatively involved in traits and processes of interest.

Despite the difficulties, genes putatively involved in SE and DNO have been identified through the search in the available databases for sequences with homology to genes associated with in vitro morphogenesis in angiosperms (reviewed in [11,12]). Recently, the complete transcriptome from different zygotic embryo developmental stages was obtained in *Pinus pinaster*, allowing a better understanding of this process in conifers and the identification of potentially relevant genes during SE [13]. Another approach consists in the comparison of material with different characteristics (e.g., material with different morphogenetic competence, responsive and non-responsive genotypes to the embryogenic or organogenic stimulus, different stages of development along the morphogenetic process…) through transcriptome and/or proteome profiling to identify differentially expressed genes. For example, Alonso et al. [14] used the suppression subtractive hybridization technique to identify genes putatively involved in the de novo shoot organogenesis in *Pinus pinea*. More recently, Rodrigues et al. [15] obtained complete small RNA libraries from different developmental stages along SE in *Pinus pinaster* in order to gain insight into the regulation of the process.

Altogether, these studies allowed the identification of genes that play key roles during SE or DNO in conifers, which were related with processes such as the regulation of the endogenous content and distribution of different PGRs, stress responses, stem cell regulation or cell wall remodeling (for a complete review, see [16,17,18,19,20]). Among them, it was reported the relevance in these processes of *WOX* and *KNOX* gene families that belong to the homeobox gene superfamily. Homeobox genes are present in all major eukaryotic lineages (invertebrates, vertebrates, plants and fungi) and encode transcriptional factors that play a key role in multiple developmental processes of multicellular organisms. They are characterized by the presence of a highly conserved region of 60 amino acids, named homeodomain, that acts as a DNA-binding domain, thereby regulating the expression of downstream target genes. Plant homeobox proteins are classified into 14 different classes: homeodomain-leucine zipper (HD-ZIP) I to IV, BEL-like (BEL), KNOTTED1-like homeobox (KNOX), plant zinc finger (PLINC), WUSCHEL-related homeobox (WOX), plant homeodomain (PHD) finger, DDT, Nodulin Homeobox genes (NDX), Luminidependens (LD), SAWADEE and Plant Interactor Homeobox (PINTOX) [21].

In this review, we show the available information about the expression pattern of homeobox genes from the *WOX* and *KNOX* gene families across SE and DNO in conifers, with the aim of elucidating their role in the molecular bases of both developmental processes. Previously, a briefly description of cellular events that occur throughout SE and DNO, and the advantages and limitations of these techniques, is presented.

## 2. In Vitro Plant Regeneration in Conifers

The two main micropropagation methods for plant regeneration are SE and DNO. Somatic embryogenesis is defined as the formation of embryos (bipolar structures containing both shoot and root meristems) from somatic cells in a process similar to zygotic embryogenesis. For its part, DNO usually involves the induction of de novo adventitious shoots on primary explants (shoot organogenesis or caulogenesis), which are subsequently excised and rooted to form plantlets (root organogenesis or rhizogenesis).

In conifers, SE was reported for the first time in 1985 in *Picea abies* [22,23] and *Larix decidua* [24]. Nowadays, there are SE and DNO protocols for multiple coniferous species, mainly for the Pinaceae family. Somatic embryogenesis is usually the preferred method for clonal propagation in conifers, but DNO can be used for species recalcitrant to SE or when this means a higher plant yield. Despite both techniques offering advantages for mass vegetative propagation, they have limitations such as SE and DNO are mainly achieved using juvenile material as explants (reviewed in [25,26,27]). Furthermore, stress during in vitro culture can cause permanent or reversible changes in explants such as chromosomal rearrangements, sequence changes in genes relevant for regeneration, alterations of the ploidy level, epigenetic changes or the activation of transposable elements, resulting in regenerated plants that are not true-to-type from their donor plant (for a complete review see [5,28]). Moreover, this so-called somaclonal variation can also affect regeneration rates and cause the loss of desirable characteristics, with the subsequent economic impact.

In the following, we will briefly describe both SE and DNO developmental processes before explaining the role of the *WOX* and *KNOX* gene families in their molecular regulation.

### 2.1. Somatic Embryogenesis in Conifers

**Figure 1:**
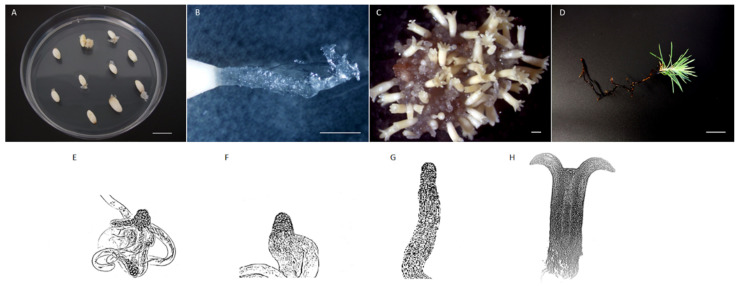
Somatic embryogenesis steps in *Pinus pinaster*. (**A**) Initiation of embryogenic cultures from immature zygotic embryos enclosed within the megagametophyte and cultured on a medium containing 2,4—dichlorophenoxyacetic acid and N^6^—benzyladenine. (**B**) Embryogenic masses (EMs) protruding through the micropyle. (**C**) Late development and maturation of cotyledonary somatic embryos achieved through the removal of plant growth regulators (PGRs), the increase in the sucrose and gelling agent concentrations, and the addition of abscisic acid (ABA). (**D**) Germination and acclimatization of plantlets. (**E**–**H**) Representation of different developmental stages across embryo differentiation. The absence of PGRs triggers the differentiation of EMs (**E**) into the early embryos (**F**) and, subsequently, into the late embryos (**G**), which have a translucent embryo proper in the apical part and an elongated suspensor in the basal part. Afterwards, reduction in water availability and ABA treatment promotes the formation of cotyledonary embryos and their maturation. Mature embryos (**H**) are prominent and opaque embryos proper, with a manifest procambium, a well-established shoot apical meristem surrounded by a whorl of cotyledons and a well-defined root apical meristem. The suspensor cells disappear as a result of programmed cell death during late differentiation. Bar 1 cm (**A**,**D**), 1 mm (**B**,**C**). Source: unpublished images from the authors.

Somatic embryogenesis in conifers is a multistage process that comprises the following steps: initiation of embryogenic cultures from explants, proliferation or multiplication of embryogenic masses (EMs), development and maturation of cotyledonary somatic embryos from EMs, germination and plantlet acclimatization (Figure 1) (for a complete review, see [16,20,26,29]).

Somatic embryogenesis is mainly achieved from mature zygotic embryos in species with simple polyembryony and from immature zygotic embryos (enclosed within the megagametophyte) in species having cleavage polyembryony [20]. However, the initiation of embryogenic cultures from mature vegetative explants in conifers is still much more challenging. This is one of the main limitations of SE, as it is only possible to evaluate plant performance during the adult vegetative or reproductive growth phases, but not during the embryonic or juvenile state, so the initial material has an unknown potential interest. The development of SE protocols using material from adult trees would allow the multiplication of trees with assessed performance, and it would reduce the required time to obtain superior varieties considerably [16]. So far, successful initiation of embryogenic cultures from adult trees was reported in a few cases, for example from needles excised from 3-year-old plants in *Picea abies* [30]; from primordial shoots in *Picea abies* [31], *Picea glauca* [32], *Pinus kesiya* [33], *Pinus patula* [34,35], *Pinus roxburghii* [36] and *Pinus wallichiana* [37]; and from secondary needles in *Pinus roxburghii* [38]. Over the last years, an international project was set with the purpose of obtaining SE from primordial shoots in six *Pinus* species with high economic importance: *Pinus contorta*, *Pinus patula*, *Pinus pinaster*, *Pinus radiata*, *Pinus strobus* and *Pinus sylvestris* [39].

Primary explants are cultured on the initiation medium, which is usually supplemented with 2,4-dichlorophenoxyacetic acid (2,4-D), a PGR with auxin activity, and the cytokinin N^6^-benzyladenine (BA), although other PGRs can be used. In addition, the initiation can take place in the absence of PGRs in some cases. Incubation on this medium gives rise to proliferating EMs, which are soft, mucilaginous and translucent to white cell aggregates. They are also characterized by the presence of small embryogenic heads, constituted by spherical and dense cells, and long vacuolated cells. For immature zygotic embryos, it is typical that EMs protrude through the micropyle (Figure 1A,B). The success of initiation varies extraordinarily depending on the species, such as Lelu-Walter et al. [16] summarized for several pine species. Once initiated, the EMs are separated from the surrounding tissue and subcultured onto maintenance medium under similar culture conditions every two or three weeks.

Prolonged serial subcultures of EMs during the multiplication phase negatively affect the number of somatic embryos obtained, the further germination of embryos and the genetic stability of cells, which can produce somaclonal variation [40,41]. A solution to avoid these problems is to cryopreserve the embryogenic lines within the first 2–4 months after initiation. This allows the conservation of EMs until field tests of trees regenerated from somatic embryos are finished. Direct cryopreservation of EMs was successfully achieved in several species (reviewed in [26]), but the most usual practice is to enclose the embryos with a chemical cryoprotectant. Some of these compounds, like dimethyl sulfoxide, might cause abnormalities in embryogenic lines. Therefore, it is recommended to assess the genetic stability of the embryogenic lines recovered and the emblings (seedlings obtained from somatic embryos) [5,42]. Apart from cryopreservation, other methods have been developed to prevent aging of EMs, such as initiation of secondary SE from cotyledonary embryos or the use of alternative culture conditions (reviewed in [16]).

The development of cotyledonary somatic embryos from EMs includes embryo differentiation and maturation (Figure 1C). The first requires the withdrawal of the PGRs used during proliferation, so the EMs are cultured on a PGR-free medium for 1–2 weeks. During differentiation, the embryo goes through several developmental stages (early, late and cotyledonary embryo), which have been well documented in *Picea abies* [7,43] and *Pinus pinaster* [44], the latter shown in Figure 1E–H. One difference between both species is that embryos of *Picea abies* differentiate in a very synchronous way, while several developmental stages can be distinguished at the same time in *Pinus pinaster*. Somatic embryos complete their differentiation and undergo maturation when they are cultured in a medium with higher concentration of gelling agent and carbohydrate, and with osmotic agents such as polyethylene glycol. All these factors reduce the water availability for embryos, promoting the growth arrest, the accumulation of storage reserves and the acquisition of desiccation tolerance [20]. The addition of abscisic acid also improves somatic embryo maturation, an essential process for proper germination of conifers embryos. A compilation of maturation medium formulation for different pine species can be found in Lelu-Walter et al. [16]. Finally, cotyledonary mature somatic embryos are germinated for obtaining plantlets that will be acclimatized in the greenhouse before transference to field (Figure 1D).

In summary, SE has several advantages compared to other vegetative propagation techniques. First, it is the most effective method for mass propagation in many coniferous species, and in some cases it can be automated for large-scale production, reducing costs and handing [26]. It also offers the highest genetic gain due to the fact that cryopreservation of embryogenic material allows the selection of superior lines prior to mass production [26]. Furthermore, embryogenic cultures can be used for gene editing and genetic transformation mediated by *Agrobacterium tumefaciens*, allowing the regeneration of trees with improved characteristics. However, SE also has limitations. Some species are either recalcitrant to plant multiplication through this technique, or their initiation rates are very low, which is common when mature zygotic embryos are used as initial explants [45]. As we mentioned before, initiation is limited to tissues from embryos or juvenile plants for most coniferous species, so it would be desirable to develop or improve protocols using material from adult trees, whose performance has been already assessed, as initial explants. Another major bottleneck of SE is the conversion of EMs into plants, due to the low rates of maturation, poor quality of the somatic embryos and low germination frequencies observed in certain species. It must be also taken into account that there is a great influence of parental genotypes on initiation rates and other stages of the SE such as maturation or recovering embryogenic lines from cryopreservation, which limits the genotype availability for micropropagation via SE.

### 2.2. De Novo Organogenesis in Conifers

Micropropagation via DNO typically begins with the differentiation of adventitious shoots on primary explants, a process that occurs through three stages. The first is the acquisition of morphogenetic competence, which is frequently associated with some level of cellular dedifferentiation. The other two stages consist of the specification of cell identity for shoot formation in response to the organogenic stimulus (induction phase), and the adventitious shoot development in the absence of that stimulus [46].

The initial explants most commonly used in DNO are complete mature zygotic embryos or parts thereof such as isolated cotyledons. In these cases, DNO is generally a direct process, as both types of explants are competent per se to respond to caulogenic stimulus without a previous dedifferentiation or callus phase [8,47]. Nevertheless, DNO can also be achieved from needle fascicles, dormant shoot buds or apical meristems. The induction medium is usually supplemented with cytokinins, being BA the most used, because it has been proven that cytokinins alone are sufficient to induce caulogenesis [48]. For each species, it is necessary to determine the optimal type and concentration of cytokinins; the minimum time of explant incubation on induction medium to elicit shoot formation (minimum induction period), which marks the onset of determination; and the period of cytokinin exposure that provides the maximal response, as longer incubation times will not enhance caulogenesis. For example, Cuesta et al. [8] obtained response after only 6 h of incubation of *Pinus pinea* cotyledons on induction medium supplemented with 44.4 µM BA, and maximal response was obtained after 2–4 days [49]. The effectiveness of DNO is determined by parameters such as the percentage of shoot-forming explants and the average number of adventitious shoots formed per explant.

The organogenic response is dependent on genotype and tissue differentiation of primary explants. In *Pinus pinea*, an important variability in caulogenic response of cotyledons from six half-sibling families was found [50], and differences were associated with the endogenous cytokinin content of cotyledonary explants throughout the organogenic process [51]. On the other hand, cotyledons excised from germinated embryos during 2, 4 and 6 days showed a loss of competence compared with those excised from non-germinated embryos [52]. Embryo germination caused a reduction in the number of buds per cotyledon, which were exclusively localized in its basal part. This effect was related to a reduction of the endogenous levels of active cytokinins and the auxin indole-3-acetic acid (IAA). It might also be a consequence of tissue differentiation, a decrease in the sensitivity to exogenous BA, and/or a decrease in BA uptake caused by the presence of waxes on the surface of precultured cotyledons [53]. Similarly, the pre-culture of *Pinus strobus* embryos on basal medium for 2 days prior to the induction caused a significant reduction in the caulogenic response [54]. However, some exceptions have been reported, as pre-culture of *Pinus radiata* seeds for 7 days enhanced the caulogenic response [55].

**Figure 2:**
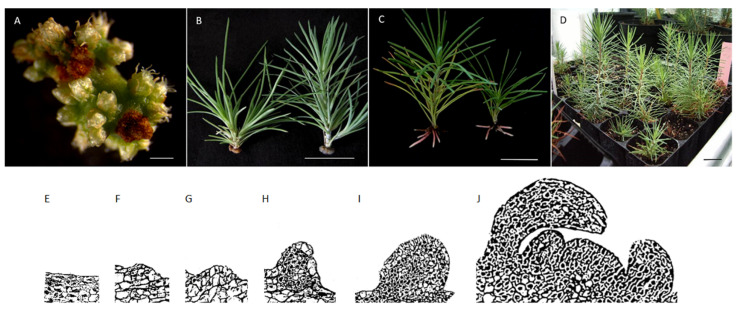
De novo organogenesis steps in *Pinus pinaster*. (**A**) Meristemoids formed on cotyledons excised from mature embryos that were cultured on the presence of N^6^—benzyladenine and subsequently transferred into a medium without plant growth regulators (PGRs). (**B**) Elongated adventitious shoots. (**C**) Rooted shoots obtained after the culture of the adventitious shoots in a medium containing 1-naphthalene acetic acid and their subsequent transference into a PGR-free medium. (**D**) Plantlets growing in the greenhouse for acclimatization. (**E**–**J**) Representation of the de novo meristem formation process, from promeristemoids to meristemoids forming needle primordia. Incubation of explants (**E**) on induction medium results in the formation of promeristemoids (**F**–**I**), which are cell clusters located within the first subepidermal cell layers of explants. They constitute the precursors of meristemoids (**J**), groups of small dense cells that arise in the explant and are determined to form adventitious shoot primordia when explants are transferred to a PGR-free medium. Bar 1 mm (**A**), 1 cm (**B**–**D**). Source: unpublished images from the authors.

After the induction phase, explants are transferred to the expression medium without PGRs, where meristemoids give rise to the formation of adventitious shoots (Figure 2A,B). Elongated shoots are then isolated and cultured firstly on root initiation medium, which is supplemented with auxins, and subsequently on root expression medium in the absence of PGRs. In *Pinus radiata*, it was reported that indole-3-butyric acid (IBA) is more efficient than 1-naphthalene acetic acid (NAA) for plant production [48], although NAA has been routinely used for adventitious root formation on *Pinus pinea* microshoots [56]. Once rooting is finished (Figure 2C), regenerated plantlets are ready for acclimatization prior to transference to field (Figure 2D). Rooting is considered one of the main bottlenecks of this technique, as very low rooting rates were obtained for some species, and a high dependence on the seed genotype was observed.

Plant multiplication via indirect organogenesis has also been reported in some coniferous species such as *Pinus taeda* [57], *Pinus radiata* [58] and *Pinus strobus* [59]. In all cases, organogenesis was achieved by culturing mature zygotic embryos in a medium for the formation of morphogenetic calli. The combination of PGRs and their concentrations used varies extraordinarily among species. In *Pinus taeda*, a high rate of callus initiation was reached adding 10 mg L^−1^ NAA and 4 mg L^−1^ BA [57] to the medium, whereas 2,4-D, NAA and IAA alone were able to induce callus formation in *Pinus strobus* [59]. In *Pinus radiata*, nodular calli were initiated from explants on medium only containing BA, but efficient proliferation took place in other supplemented with BA and IBA [58]. After proliferation, calli are transferred to the organogenic induction medium, which usually contains auxin and cytokinin at a certain proportion, for differentiating adventitious buds. Then, buds are elongated and finally rooted. Tang and Newton [59] demonstrated that treatment of calli at 4 °C for 6 weeks improved the yield of the process. Furthermore, the addition of putrescine to the media decreased callus browning and improved callus formation, adventitious bud formation and rooting rates, as this polyamine reduces lipid peroxidation.

Compared to SE, DNO from zygotic embryos have the disadvantage that there are no effective long-term cryopreservation methods to maintain the juvenility of the material until field trials are finished, with few exceptions (reviewed in [26]). The development of effective cryopreservation protocols or appropriate genetic markers would allow within-family selection of superior genotypes, and organogenesis would become as effective as SE in achieving genetic gain [4,26]. In spite of this inconvenience, DNO is used when their effectiveness is higher than that of SE, as it happens in *Pinus pinea*. In this specie, only around 0.5% of initial zygotic embryos produce established embryogenic lines [60] whereas at least 70 plantlets per seed can be produced at optimal conditions through organogenesis [56]. Somatic embryogenesis and DNO may be also used together, which is particularly useful when maturation and germination rates of somatic embryos are very low, especially in genetically transformed lines. Montalbán et al. [61] reported that each somatic embryo in *Pinus radiata* can form around 19 adventitious shoots, with a rooting rate of 60%. Alvarez et al. [41] also found axillary shoot formation after the culture of *Pinus pinaster* mature somatic embryos in the presence of 10 µM BA for 7 days, which could be isolated and rooted, increasing the yield of SE.

One advantage of DNO against SE is the possibility to regenerate plants using explants derived from adult selected genotypes and appropriate protocols (reviewed in [62]). Thus, Cortizo et al. [63] reported shoot initiation in brachyblast primordia from winter-dormant buds collected from 20–25 year-old trees in *Pinus pinea*. In particular, the buds without scales were sectioned into slices of 0.5–1 cm in thickness and cultured on a medium with 2.5 µM of thidiazuron, a synthetic compound with cytokinin activity. After that, the explants were transferred to a PGR-free elongation medium for the development of the microshoots. When these reached approximately 1 cm, they were isolated, elongated and eventually rooted (adventitious roots). The downsides of this protocol are the high influence of the donor genotype in the response and the low rooting rates obtained, which suggest that this method induced reinvigoration instead of rejuvenation. Similar protocols have been described for adult trees of *Pinus pinaster* [64] and *Pinus sylvestris* [65]. The difference was that the elongated needle fascicles were excised and cultured again on initial medium to promote axillary bud proliferation. In *Pinus pinaster*, high organogenic response was achieved with 25 µM zeatin and meta-topolin, but only those shoots obtained under 25 µM BA were able to develop properly and form adventitious roots. Multiplication of adult trees can also be achieved through the culture of apical meristems. Another alternative strategy is the introduction of adult material in vitro via microblast micrografting in seedling rootstocks [66].

## 3. The Role of *WOX* Genes during Somatic Embryogenesis and De Novo Organogenesis in Conifers

**Figure 3:**
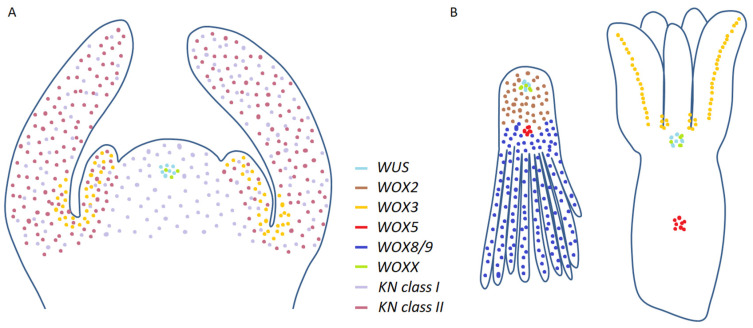
Schematic representation of the expression domains of some *WOX* and *KNOX* genes in conifers according to quantitative real-time PCR, RNA sequencing RNA-seq and in situ mRNA hybridization results. (**A**) Shoot apex; (**B**) late and mature somatic embryo. Source: unpublished drawings from the authors.

*WOX* genes constitute a plant-specific homeobox family whose members have important functions during plant growth and development, such as embryo patterning, organ formation and stem cell maintenance. Phylogenetic analyses carried out by van der Graaff et al. [67] have established three distinct clades in the *WOX* gene family: the ancient clade, whose members are present in all plant lineages from green algae to seed plants; the intermediate clade, present in vascular plants; and the modern or WUS clade, only found in ferns and seed plants. The *WOX* gene family includes 14 members in *Pinus pinaster* and 13 in *Picea abies* distributed throughout the three clades previously mentioned [68,69]. The analysis of their expression during SE and in different plantlet tissues by quantitative real-time PCR (RT-qPCR), RNA sequencing and in situ mRNA hybridization showed that the expression profiles of *WOX* genes in conifers are quite similar to those described for their angiosperm counterparts (Figure 3), suggesting a high degree of conservation of the gene family across seed plants [68,69]. *WOX* gene family diversity in *Arabidopsis thaliana* and several gymnosperm species are presented in more detail in Table 1 at the end of this section.

Ancient-clade genes are constitutively expressed in all developmental stages of SE but also in all plantlet tissues analyzed in *Picea abies* and *Pinus pinaster* [68,69] (see Supplementary Figure S1), which is consistent to what was previously reported in angiosperms [70], although their function in conifers still remains unknown. In contrast, the WUS-clade member *WOX2* and most members from the intermediate clade are mainly expressed during early and late SE, with low expression levels in mature somatic embryos, both in *Picea abies* and *Pinus pinaster* [68,69]. Besides, expression of *PaWOX2* was also detected by in situ mRNA hybridization in immature zygotic embryos in *Picea abies*, but not in the mature ones [71]. However, practically no expression was found during zygotic embryo germination or in plantlets for *WOX2* and most intermediate members in the analyzed coniferous species. Based on this expression pattern, *WOX2* has been proposed as a good marker of early stages of SE in *Picea abies* [72,73]. For example, *WOX2* allowed distinguishing EMs from non-embryogenic calli during SE from primordial shoots in *Picea glauca* [32]. Similarly, this gene was only expressed in EMs derived from shoots buds and immature zygotic embryos, but not in non-embryogenic callus induced from young needles of 1-month-old seedlings in *Pinus contorta* [74].

Orthologues of these genes in *Arabidopsis thaliana*, *AtWOX2* and the members from the intermediate clade *AtWOX8* and *AtWOX9* are involved in early embryonic pattern formation [75,76]. Basically, *AtWOX2* and *AtWOX8* are expressed in the female gametophyte and zygote. After the first division *AtWOX2* transcripts are only detected in the apical daughter cell that will originate the embryo proper, while *AtWOX8* expression is restricted to the basal daughter cell that will give rise to the embryo suspensor and the hypophyseal cell, establishing in that way the apical-basal polarity of the embryo. For its part, *AtWOX9* also contributes to the embryo polarity, as it is expressed initially in the hypophysis and then expands into the central domain of the embryo. In *Picea abies*, *PaWOX2* and the intermediate-clade member *PaWOX8/9* have been also shown to participate in the establishment of the apical-basal embryo pattern during early embryo development [71,77]. In order to unravel their role in this process, RNA interference (RNAi) lines for each gene were constructed using both constitutive and inducible promoters. Downregulation of *PaWOX2* and *PaWOX8/9* through RNAi during the first stages of SE results in aberrant embryos due to the lack of a well-defined border between the globular EM and the suspensor, failing to form mature somatic embryos at a higher frequency than the control lines. In both cases, the effects of inhibiting their expression are observed mainly during early embryo differentiation, and practically no defects were observed when downregulation takes place after late embryo formation. In the case of *PaWOX8/9*, an alteration of the cell division planes in the basal cells of the EM, and the differentiation of suspensor cells (both basal and top cells), was observed by confocal microscopy [77]. In fact, it was reported that *PaWOX8/9* RNAi lines showed altered expression levels of several cell-cycle-regulating genes. Whereas *PaWOX8/9* regulates cell division at the transcriptional level and cell fate determination, downregulation of *PaWOX2* does not affect the expression of the genes that participate in the regulation of the cell cycle [71]. Instead of that, high expression levels of *PaWOX2* are required during early embryogenesis for the correct development of the protoderm, the external layer of the globular embryo which will give rise to the epidermis, in early and late embryos. Furthermore, this gene has been shown to be essential for the expansion of the suspensor cells during early embryo development. Other members from the intermediate clade in conifers are phylogenetically close to *AtWOX11* and *AtWOX12*, which have been related to root organogenesis [78], although no information about their role in conifers is still available.

The WUS clade in conifers contains orthologues of the genes *WUS*, *WOX5*, *WOX3* and *WOX4* previously described in angiosperms [68,69]. In *Arabidopsis thaliana*, these genes have been involved in the maintenance of stem cells in the SAM, root apical meristem (RAM), leaf marginal meristems and procambium, respectively [79,80,81,82] (see Supplementary Figure S1). However, no orthologues have been found for *AtWOX1*, *AtWOX6* and *AtWOX7*, which have been shown to participate in lateral organ primordia formation, cold-stress responses and lateral root development, respectively [83,84,85,86].

In conifers, *WUS* expression is low during the first stages of SE and reaches a peak in somatic mature embryos, when the SAM is already established [68,69]. In 3-week-old plantlets, transcripts were detected exclusively in a small group of cells situated in the central zone of the SAM through RT-qPCR and in situ mRNA hybridization [69], which might indicate that *PpWUS* regulates the balance between proliferation and differentiation of stem cells, similarly to what was established in angiosperms. Interestingly, the effects of inducible ectopic expression of *AtWUS* were analyzed in different stages of SE, germinating somatic embryos and seedlings in *Picea glauca* [87]. Expression of *AtWUS* caused important alterations during somatic embryo formation. In germinating embryos, induction of *AtWUS* expression inhibited root growth, but normal shoot development was observed, supporting the participation of this gene in SAM maintenance. In contrast to *Arabidopsis thaliana*, expression of *AtWUS* did not induce ectopic shoot formation on *Picea glauca* seedlings. It is noticeable that the WUS clade in gymnosperms contains a gene absent in angiosperms called *WOXX,* whose expression profile during SE and in plantlets in *Pinus pinaster* is similar to that described for *PpWUS* [69,88].

On the other side, analyses of conifer *WOX3* orthologues suggest their involvement in lateral organ formation and differentiation, but not in meristem formation. Expression of *PaWOX3* was very low during early and late embryogenesis in *Picea abies*, reaching its highest value in mature somatic embryos [89]. In particular, these authors detected *PaWOX3* expression at the base and lateral margins of cotyledons from mature embryos through in situ mRNA hybridization and GUS staining in pPaWOX3:GUS lines. Furthermore, downregulation of *PaWOX3* through RNAi did not affect somatic embryo formation, but alters their cotyledon morphology. In three-week-old plantlets of *Pinus pinaster*, Alvarez et al. [69] detected *PpWOX3* transcripts in lateral organs and in the peripheral zone of the SAM, where organ initiation takes place (see Figure 3B and Supplementary Figure S1).

Before *WUS* functionality in the SAM was established, some authors proposed that *WOX5* regulated stem cell maintenance both in the SAM and RAM in conifers [68,90]. This hypothesis was based on the fact that *WOX5* transcripts were detected by RT-qPCR mainly in root apexes but also in shoot apexes in several coniferous species, whereas no *WUS* expression was detected in any tissues or developmental stages at that moment. However, as we mentioned before, recent studies have determined that *WUS* and *WOX5* exert their functions of stem cell regulators in the SAM and RAM, respectively, in conifers [69]. Although current evidence support that the functional differentiation of *WUS* and *WOX5* took place before the gymnosperm–angiosperm split, it cannot be discarded an additional role of *WOX5* in conifer SAM functioning based on its expression pattern during SE and in plantlets (see Supplementary Figure S1). Similar to *WUS*, *WOX5* also reaches maximum expression levels during SE in mature embryos in *Picea abies* and *Pinus pinaster*, and expression of this gene was also detected in shoot apexes of plantlets [68,69]. In addition, recent interspecies complementation experiments have shown that the expression of both *WUS* and *WOX5* orthologues from different gymnosperm species under the control of *AtWUS* and *AtWOX5* promoters can rescue the phenotypes of the *Arabidopsis wus-1* and *wox5-1* loss-of-function mutants [91]. These findings suggest that gymnosperm WUS and WOX5 proteins are interchangeable when expressed under the right conditions, as it had been previously established in angiosperms [92].

Based on these results, Alvarez et al. [93] analyzed the expression pattern of *PpWUS*, *PpWOXX* and *PpWOX5* during the induction phase of in vitro caulogenesis in *Pinus pinea* to determine their participation in de novo shoot meristem formation. In particular, transcript levels of these genes, among others, were measured in *Pinus pinea* cotyledons cultured on the presence and absence of 44.4 µM BA during short and long times of culture (0–1 d and 2–6 d, respectively) and analyzed by principal component analysis. The authors found that no *PpWOXX* expression was detected along the process, whereas *PpWUS* seems to have an important role at long times of induction. In *Arabidopsis thaliana*, it was also reported that cytokinin signaling eventually lead to the upregulation of *WUS* during the induction phase of de novo shoot organogenesis in the center of the incipient shoot meristem [94,95,96]. Expression data were also analyzed in *Pinus pinea* cotyledons together with the endogenous content of several PGRs by partial least squares regression. Results reinforced the participation of *PpWUS* in the organogenic induction at long times of culture, but also pointed out that *PpWOX5* has a relevant participation in this process, although its exact role still remains unknown.

**Table 1:** List of genes belonging to the *WUSCHEL-RELATED HOMEOBOX* (*WOX*) family, including those from model species *Arabidopsis thaliana* and their homologue genes already identified in gymnosperms, with name abbreviation, locus code (AGI code in case of *Arabidopsis thaliana*, GenBank number in case of gymnosperm species), function, location and references. Shoot apical meristem, SAM; root apical meristem, RAM.

Species	Name Abbreviation	Locus Code	Function and Location	References
***i. WUS clade***				
*Arabidopsis thaliana*	*AtWOX1*	AT3G18010	Lateral organ primordia formation	[75,84,85]
	*AtWOX2*	AT5G59340	Apical embryo and embryo patterning	[75,76]
	*AtWOX3/PRS*	AT2G28610	SAM, lateral organ formation	[81]
	*AtWOX4*	AT1G46480	Vascular tissue, procambial development	[82]
	*AtWOX5*	AT3G11260	Stem cell maintenance (RAM)	[80]
	*AtWOX6*	AT2G01500	Cold-stress response	[83]
	*AtWOX7*	AT5G05770	Lateral root development	[86]
	*AtWUS*	AT2G17950	Stem cell maintenance (SAM)	[79]
*Ginkgo biloba*	*GbWOX2*	FM882124	Embryo patterning	[88]
	*GbWOX3A*	FM882125	Lateral organ outgrowth	[88]
	*GbWOX3B*	FM882126	Lateral organ outgrowth	[88]
	*GbWOX4*	HF564615	Germinating embryo, vascular cambium	[88]
	*GbWUS*	FM882128	Embryo, shoot tip	[88,90]
*Gnetum gnemon*	*GgWOX2A*	HF564611	Embryo patterning	[88]
	*GgWOX2B*	HF564619	Embryo patterning	[88]
	*GgWOX4*	HF564612	Germinating embryo, vascular cambium	[88]
	*GgWOX6/WOXX*	HF564620	n/a	[88]
	*GgWOXY*	HF564621	n/a	[88]
	*GgWUS*	FM882154	Embryo, shoot tip	[88,90]
*Picea abies*	*PaWOX2*	AM286747	Embryo patterning	[68,71,72,73]
	*PaWOX3*	JX411947	Lateral organ outgrowth	[68,89]
	*PaWOX4*	JX411948	Germinating embryo, vascular cambium	[68]
	*PaWOX5*	JX411949	Embryo, SAM, RAM	[68]
	*PaWOXX*	KX011459	Embryo, SAM, needles	[69]
	*PaWUS*	JX512364	Embryo, shoot tip	[68]
*Pinus pinaster*	*PpWOX2*	KU962991	Embryo patterning	[69]
	*PpWOX3*	KU962992	Lateral organ outgrowth	[69]
	*PpWOX4*	KU962993	Germinating embryo, vascular cambium	[69]
	*PpWOX5*	KT356216	Embryo, SAM, RAM	[69]
	*PpWOXX*	KU962995	Embryo, SAM, needles	[69]
	*PpWUS*	KT356213	Embryo, shoot tip	[69]
*Pinus sylvestris*	*PsWOX2*	FM882159	Embryo patterning	[90]
	*PsWOX3*	FM882158	Lateral organ outgrowth	[90]
	*PsWOX4*	HF564616	Germinating embryo, vascular cambium	[90]
	*PsWOX5/WUS*	FM882160	Embryo, SAM, RAM	[90]
*Pinus taeda*	*PtWOX2*	KX011449	Embryo patterning	[69]
	*PtWOX3*	KX011450	Lateral organ outgrowth	[69]
	*PtWOX4*	KX011451	Germinating embryo, vascular cambium	[69]
	*PtWOX5*	KX011452	Embryo, SAM, RAM	[69]
	*PtWOXX*	KX011454	Embryo, SAM, needles	[69]
	*PtWUS*	KX011458	Embryo, shoot tip	[69]
***ii. Intermediate clade***				
*Arabidopsis thaliana*	*AtWOX8/STPL*	AT5G45980	Basal embryo patterning	[75,76]
	*AtWOX9/STIMPY*	AT2G33880	Basal embryo patterning, cell proliferation	[75]
	*AtWOX11*	AT3G03660	Adventitious root formation	[78]
	*AtWOX12*	AT5G17810	De novo root organogenesis	[78]
*Ginkgo biloba*	*GbWOX9*	HF564618	n/a	[88]
*Gnetum gnemon*	*GgWOX9*	HF564613	n/a	[88]
*Picea abies*	*PaWOX8/9*	GU944670	Embryo patterning	[68,73,77]
	*PaWOX8A*	JX411950	Embryo patterning	[68]
	*PaWOX8B*	JX411951	Embryo patterning	[68]
	*PaWOX8C*	JX411952	Embryo patterning	[68]
	*PaWOX8D*	JX411953	Embryo patterning	[68]
*Pinus pinaster*	*PpWOXB*	KU962997	Embryo patterning	[69]
	*PpWOXC*	KU962998	Embryo patterning	[69]
	*PpWOXD*	KU962999	Embryo patterning	[69]
	*PpWOXE*	KU963000	Embryo patterning	[69]
	*PpWOXF*	KU963001	Embryo	[69]
*Pinus sylvestris*	*PsWOX9*	FM882155	n/a	[90]
*Pinus taeda*	*PtWOXB*	KX011456	Embryo patterning	[69]
	*PtWOXE*	KX011457	Embryo patterning	[69]
***iii. Ancient clade***				
*Arabidopsis thaliana*	*AtWOX10*	AT1G20710	n/a	[67,70]
	*AtWOX13*	AT4G35550	Floral transition, root development	[70]
	*AtWOX14*	AT1G20700	Floral transition, root development	[70]
*Ginkgo biloba*	*GbWOX13*	HF564617	n/a	[88]
*Gnetum gnemon*	*GgWOX13*	HF564614	n/a	[88]
*Picea abies*	*PaWOX13*	n/a	n/a	[68]
	*PaWOXG*	MG545153	n/a	[69]
*Pinus pinaster*	*PpWOX13*	KU962994	n/a	[69]
	*PpWOXA*	KU962996	n/a	[69]
	*PpWOXG*	MG545154	n/a	[69]
*Pinus sylvestris*	*PsWOX13*	FM882156	n/a	[90]
*Pinus taeda*	*PtWOX13*	KX011453	n/a	[69]
	*PtWOXA*	KX011455	n/a	[69]
	*PtWOXG*	MG545155	n/a	[69]

n/a: non available information.

## 4. The Role of *KNOX* Genes during Somatic Embryogenesis and De Novo Organogenesis in Conifers

*KNOX* genes constitute another plant-specific homeobox gene family whose members have been found in practically all plant lineages: green algae, bryophytes, lycophytes, ferns, angiosperms and gymnosperms. Whereas only one class of *KNOX* genes has been reported in algae, phylogenetical analyses established two different subfamilies in land plants designated class I and class II [97,98]. Recently, *KNOX* genes lacking the characteristic homeodomain were described exclusively in some dicotyledonous species, which constituted the so-called class M subfamily [99]. *KNOX* genes from class I and class II subfamilies differ in their sequence, expression patterns and function. In angiosperms, class I members are mainly expressed in meristematic regions. The *Arabidopsis thaliana* gene named *SHOOT MERISTEMLESS* (*STM*) is essential for SAM formation during embryogenesis and participates in the maintenance of the stem cell population in the center of the SAM [100,101]. Loss-of-function *stm* mutants lack a functional SAM [102,103], whereas overexpression of this gene results in the formation of ectopic meristems and lobed leaves [104], which indicates a role of *STM* in determining leaf morphology [105]. Furthermore, *STM* expression is upregulated during de novo shoot organogenesis [106]. *STM* along with other class I members like *BREVIPEDICELLUS/KNOTTED IN ARABIDOPSIS THALIANA 1* (*BP/KNAT1*) and *KNAT2* also play a key role in the development of floral meristem and carpel formation [107,108,109]. For its part, the class I member *KNAT6* is expressed during embryogenesis and participates in the establishment of the boundaries between the SAM and cotyledons [110] (see Supplementary Figure S2). On the other side, class II *KNOX* genes are expressed mainly in differentiating tissues and mature organs, and participate in organ differentiation [111] (see Supplementary Figure S2). Unlike *STM*, overexpression of class II members causes a simplification of leaf morphology in plants with complex leaves [105].

In conifers, four class I members have been described to date in several spruce and pine species, which were designated *KN1* to *KN4* [112,113,114,115]. More recently, two members from class II subfamily were isolated in *Pinus pinaster* and other coniferous species, which were designated *KN5* and *KN6* [111,115]. Studies of their expression by RT-qPCR and in situ mRNA hybridization in plantlets (Figure 3), together with analyses of their overexpression in the heterologous system *Arabidopsis thaliana*, support that the functional differentiation established in angiosperms might be evolutionarily conserved between gymnosperms and angiosperms to a great extent [115] (see Supplementary Figure S2). Function and/or expression domains of *KNOX* genes from different coniferous species and their *Arabidopsis thaliana* counterparts are summarized in Table 2 at the end of this section.

Due to the important participation of class I members in the embryogenic developmental pathway in angiosperm, particularly in meristem formation and establishment, class I members have been studied during SE and de novo shoot organogenesis in conifers in order to determine their specific role in these processes. Expression of class I *KNOX* genes was reported along the maturation phase of SE in *Picea abies* and *Pinus pinaster* [115,116]. The expression of the four class I members was analyzed in competent and non-competent embryogenic lines from *Picea abies* [113,116]. Results showed that *HBK1* and *HBK3* (here designated *PaKN2* and *PaKN1*, respectively, for convenience) expressed in both types of lines, whereas expression of *HBK2* and *HBK4* (here designated *PaKN3* and *PaKN4*, respectively, for convenience) was only detected in those lines that give rise to mature cotyledonary embryos, but not in those in which conversion of EMs to embryos is blocked. The expression profiles of these four class I genes were also analyzed in different developmental stages of *Picea abies* embryogenic lines treated and non-treated with N-1-naphthylphthalamic acid (NPA), an inhibitor of the polar auxin transport [116]. Previous studies had shown that polar auxin transport is essential for the correct formation of a functional SAM and RAM during embryogenesis, as NPA treatment gives rise to the formation of aberrant somatic embryos with fused or aborted cotyledons that lack a visible SAM, and are unable to germinate [117]. An increase in *PaKN3* and *PaKN4* expression was detected during SAM establishment in control lines, which is delayed in NPA-treated lines, suggesting that these genes are essential for the proper SAM formation during embryogenesis. On the other side, *PaKN1* and *PaKN2* expression was upregulated during the first stages of embryogenesis, and their levels were not altered by NPA treatment along the process. These results indicate that these genes have a more general role in embryo development, especially during the early phases of embryogenesis, but not in SAM establishment.

The role of *PaKN1* during embryogenesis was deeply studied in transgenic lines of *Picea abies* [118]. Overexpression of this gene accelerates the formation of early embryos from EMs, which also have bigger embryogenic heads and enlarged suspensors compared to the control, and eventually lead to the formation of mature cotyledonary embryos at a higher frequency. These embryos have similar morphology and germination rates than control ones, giving rise to viable plants with no phenotypical defects, although it is remarkable that embryos derived from *PaKN1*-overexpressing lines tend to have enlarged SAMs. In contrast, down-regulation of *PaKN1* significantly reduced differentiation of EMs into immature somatic embryos, which failed to form mature cotyledonary embryos. These results support the relevance of *PaKN1* during the first stages of embryo development, although it also has an important role during late embryogenesis. Later studies have found that *PaKN1* expression affects glutathione and ascorbate metabolism, which play a key role in embryo development [119].

Class I *KNOX* expression was also analyzed during the initiation of SE from primordial shoots in *Picea glauca* [32]. In particular, transcript levels of *SKN1*, *SKN2*, *SKN3* and *SKN4* (here designated *PgKN1* to *PgKN4* for convenience) were measured in primordial shoots after different incubation times on induction medium (0, 3 and 6 days), in EMs and in non-embryogenic tissue, among other tissues. All *PgKN* genes were already expressed in non-treated primordial shoots. In fact, *PgKN4* is expressed mainly in the initial explants and decreases with incubation time. Little *PgKN4* expression was detected in Ems, and it was undetectable in non-embryogenic tissue. For its part, *PgKN1* and *PgKN2* showed a similar expression pattern, as the highest expression of these genes was reported in Ems, and no expression was detected in non-embryogenic tissue. On the other side, *PgKN3* expresses at very high level in non-embryogenic tissue.

Results from Klimaszewska et al. [32] suggest that *KN1* and *KN2* can be used as markers during the initial steps of SE for the discrimination of EMs from non-embryogenic calli. This is not the case of *KN3*, which showed high expression in non-embryogenic calli in *Picea glauca*. However, *KN3* and *KN4* orthologues might constitute good markers for the maturation competence of embryogenic lines [116].

Based on the expression data commented above and its phylogenetic proximity, some authors proposed that *KN1* and *KN2* orthologues might perform redundant roles during early embryogeny in conifers [115,116]. Furthermore, these genes are located close to each other on the same linkage group and are thought to have arisen after a duplication event [114]. For its part, *KN3* and *KN4* seem to play a key role in SAM formation during SE in *Picea abies* [116]. It is remarkable that conifer *KN3* orthologues are phylogenetically very close to *AtSTM* [115]. Interestingly, class I *KNOX* gene expression during de novo shoot organogenesis in *Pinus pinea* was analyzed by multivariate statistics, revealing that both *PpKN2* and *PpKN3* have a relevant role during the acquisition of shoot meristem identity [93] (see Supplementary Figure S2). However, further studies are necessary to elucidate the specific role of each class I member in conifers.

**Table 2:** List of genes belonging to the *KNOTTED1-LIKE HOMEOBOX (KNOX)* family, including those from model species *Arabidopsis thaliana* and their homologue genes already identified in gymnosperms, with name abbreviation, locus code (AGI code in case of *Arabidopsis thaliana*, GenBank number in case of gymnosperm species), function, location and references. Shoot apical meristem, SAM.

Species	Name Abbreviation	Locus Code	Function and Location	References
***i. Class I***				
*Arabidopsis thaliana*	*AtSTM*	AT1G62360	SAM formation and maintenance of stem cell population, floral and carpel formation	[100,101,102]
	*AtBP/KNAT1*	AT4G08150	Stem cell maintenance	[107,108,109]
	*AtKNAT2*	AT1G70510	Carpel development	[107,108,109]
	*AtKNAT6*	AT1G23380	Establishment SAM boundaries during embryogenesis, shoot apex and root	[110]
*Picea abies*	*PaKN1/HBK3*	AF483278	General functions on somatic embryo development	[113,114,116,118,119]
	*PaKN2/HBK1*	AF063248	SAM of vegetative and reproductive buds and general functions on somatic embryos	[112,113,114,116]
	*PaKN3/HBK2*	AF483277	Embryogenic cell lines competent to form fully mature embryos	[113,114,116]
	*PaKN4/HBK4*	AY680389/AY680400	Embryogenic cell lines competent to form fully mature embryos	[114,116]
*Picea glauca*	*PgKN1*	AY680381/AY680392	n/a	[114]
	*PgKN2*	AY680383/AY680394	n/a	[114]
	*PgKN3*	AY680385/AY680396	n/a	[114]
	*PgKN4*	AY680390/AY680401	n/a	[114]
*Picea mariana*	*PmKN1*	U90091	n/a	[114]
	*PmKN2*	U90092	n/a	[114]
	*PmKN3*	AY680386/AY680397	n/a	[114]
	*PmKN4*	AY680405	n/a	[114]
*Pinus pinaster*	*PpKN1*	KT356208	Embryo, hypocotyl, root and shoot apex	[115]
	*PpKN2*	KT356209	Somatic embryo and germination	[115]
	*PpKN3*	KT356217/KT356211	SAM and vascular tissues, hypocotyl and shoot apex	[115]
	*PpKN4*	KT356210	Embryo, hypocotyl, root and shoot apex	[115]
*Pinus strobus*	*PsKN1*	AY680380/AY680391	n/a	[114]
	*PsKN2*	AY680382/AY680393	n/a	[114]
	*PsKN3*	AY680384/AY680395	n/a	[114]
	*PsKN4*	AY680388/AY680399	n/a	[114]
*Pinus taeda*	*PtKN1*	AY680402	n/a	[114]
	*PtKN2*	AY680403	n/a	[114]
	*PtKN3*	AY680404	n/a	[114]
	*PtKN4*	AY680387/AY680398	n/a	[114]
***ii. Class II***				
*Arabidopsis thaliana*	*AtKNAT3*	AT5G25220	Mature organs	[111]
	*AtKNAT4*	AT5G11060	Mature organs	[111]
	*AtKNAT5*	AT4G32040	Mature organs	[111]
	*AtKNAT7*	AT1G62990	Mature organs	[111]
*Picea abies*	*PaKN5*	MK580154	n/a	[115]
*Pinus pinaster*	*PpKN5*	MK580155	Shoot apex and primordia of young needles	[115]
	*PpKN6*	MK580156	Early embryos	[115]
*Pinus taeda*	*PpKN5*	MK580157	n/a	[115]
	*PpKN6*	MK580158	n/a	[115]
***iii. Class M***				
*Arabidopsis thaliana*	*AtKNATM*	AT1G14760	Lateral domain on flower meristem, involved on flower transition	[98,99]

n/a: non available information.

## Data Availability

Publicly available datasets were analyzed in this study. Sequences used can be found at https://www.ncbi.nlm.nih.gov/genbank/ (accessed on 18 March 2021).

## Supplementary Materials

The following are available online at https://www.mdpi.com/2073-4425/12/3/438/s1. Supplementary Figure S1. Comparison of tissue expression of the most relevant genes from *WOX* family of *Arabidopsis thaliana* and *Pinus pinaster* within different tissues and developmental stages. The selected genes belong to (i) WUS clade, such as *WOX3* gene in both Arabidopsis (**A**) and *Pinus pinaster* (**B**), sharing common patterns on SAM; and *WUS* gene in Arabidopsis (**C**) and *WOX5* gene in *Pinus pinaster* (**D**) with distinct expression pattern; (ii) intermediate clade, such as *WOX9* gene in Arabidopsis (**E**) and *WOXE* gene in *Pinus pinaster* (**F**), with common root expression patterns; and (iii) ancient clade, such as *WOX13* gene in both Arabidopsis (**G**) and *Pinus pinaster* (**H**), with shared expression patterns on SAM but specific RAM expression in *Pinus pinaster*. Developmental map from *Arabidopsis thaliana* comes from Arabidopsis eFP Browser, in case of *Pinus pinaster* developmental map comes from the exImage tool at ConGenIE.org (http://v22.popgenie.org/microdisection/ (accessed on 18 March 2021)). Supplementary Figure S2. Comparison of tissue expression of the most relevant genes from KNOX family of *Arabidopsis thaliana* and *Pinus pinaster* within different tissues and developmental stages. The selected genes belong to (i) class I, such as *KNAT6* gene in Arabidopsis (**A**) and *KN2* gene in *Pinus pinaster* (**B**); and (ii) class II, such as *KN3* gene in Arabidopsis (**C**) and *KN5* gene in *Pinus pinaster* (**D**). Developmental map from *A. thaliana* comes from Arabidopsis eFP Browser, in case of *Pinus pinaster* developmental map comes from the exImage tool at ConGenIE.org (http://v22.popgenie.org/microdisection/ (accessed on 18 March 2021)).

## Abbreviations

Abscisic acid, ABA; N^6^-benzyladenine, BA; 2,4-dichlorophenoxyacetic acid, 2,4-D; de novo organogenesis, DNO; embryogenic mass, EM; indole-3-acetic acid, IAA; indole-3-butyric acid, IBA; *KNOTTED1-LIKE HOMEOBOX*, *KNOX*; 1-naphthalene acetic acid, NAA; N-1-naphthylphthalamic acid, NPA; plant growth regulator, PGR; quantitative real-time PCR, RT-qPCR; RNA interference, RNAi; root apical meristem, RAM; shoot apical meristem, SAM; somatic embryogenesis, SE; *WUSCHEL-RELATED HOMEOBOX*, *WOX.*
