# Supplementary material for: In Vitro Plant Regeneration in Conifers: The Role of WOX and KNOX Gene Families
Source: Genes (Basel). 2021 Mar 19;12(3):438. doi: 10.3390/genes12030438 (PMC8003479; doi:10.3390/genes12030438)

Supplementary Figure Legends:

Supplementary Figure S1. Comparison of tissue expression of the most relevant genes from WOX family of *Arabidopsis thaliana* and *Pinus pinaster* within different tissues and developmental stages. The selected genes belong to (i) WUS clade, such as *WOX3* gene in both Arabidopsis (A) and *Pinus pinaster* (B), sharing common patterns on SAM; and *WUS* gene in Arabidopsis (C) and *WOX5* gene in *Pinus pinaster* (D) with distinct expression pattern; (ii) intermediate clade, such as *WOX9* gene in Arabidopsis (E) and *WOXE* gene in *Pinus pinaster* (F), with common root expression patterns; and (iii) ancient clade, such as *WOX13* gene in both Arabidopsis (G) and *Pinus pinaster* (H), with shared expression patterns on SAM but specific RAM expression in *Pinus pinaster*. Developmental map from *Arabidopsis thaliana* comes from Arabidopsis eFP Browser (Winter et al. 2017), in case of *Pinus pinaster* developmental map comes from the exImage tool at ConGenIE.org (http://v22.popgenie.org/microdisection/) (Cañas et al. 2017).

Supplementary Figure S2. Comparison of tissue expression of the most relevant genes from KNOX family of *Arabidopsis thaliana* and *Pinus pinaster* within different tissues and developmental stages. The selected genes belong to (i) class I, such as *KNAT6* gene in Arabidopsis (A) and *KN2* gene in *Pinus pinaster* (B); and (ii) class II, such as *KN3* gene in Arabidopsis (C) and *KN5* gene in *Pinus pinaster* (D). Developmental map from *A. thaliana* comes from Arabidopsis eFP Browser (Winter et al. 2017), in case of *Pinus pinaster* developmental map comes from the exImage tool at ConGenIE.org (http://v22.popgenie.org/microdisection/) (Cañas et al. 2017).

References:

Cañas, R.A.; Li, Z.; Pascual, M.B.; Castro-Rodríguez, V.; Ávila, C.; Sterck, L.; Van de Peer, Y.; Cánovas, F.M. The gene expression landscape of pine seedling tissues. *Plant Journal.* **2017**, *91(6),* 1064–1087. https://doi.org/10.1111/tpj.13617

Winter, D.; Vinegar, B.; Nahal, H.; Ammar, R.; Wilson, G.V.; Provart, N.J. An "Electronic Fluorescent Pictograph" Browser for Exploring and Analyzing Large-Scale Biological Data Sets. *PLOS ONE*. **2007**, *2(8)*: e718. https://doi.org/10.1371/journal.pone.0000718

Suppl. Figure 1

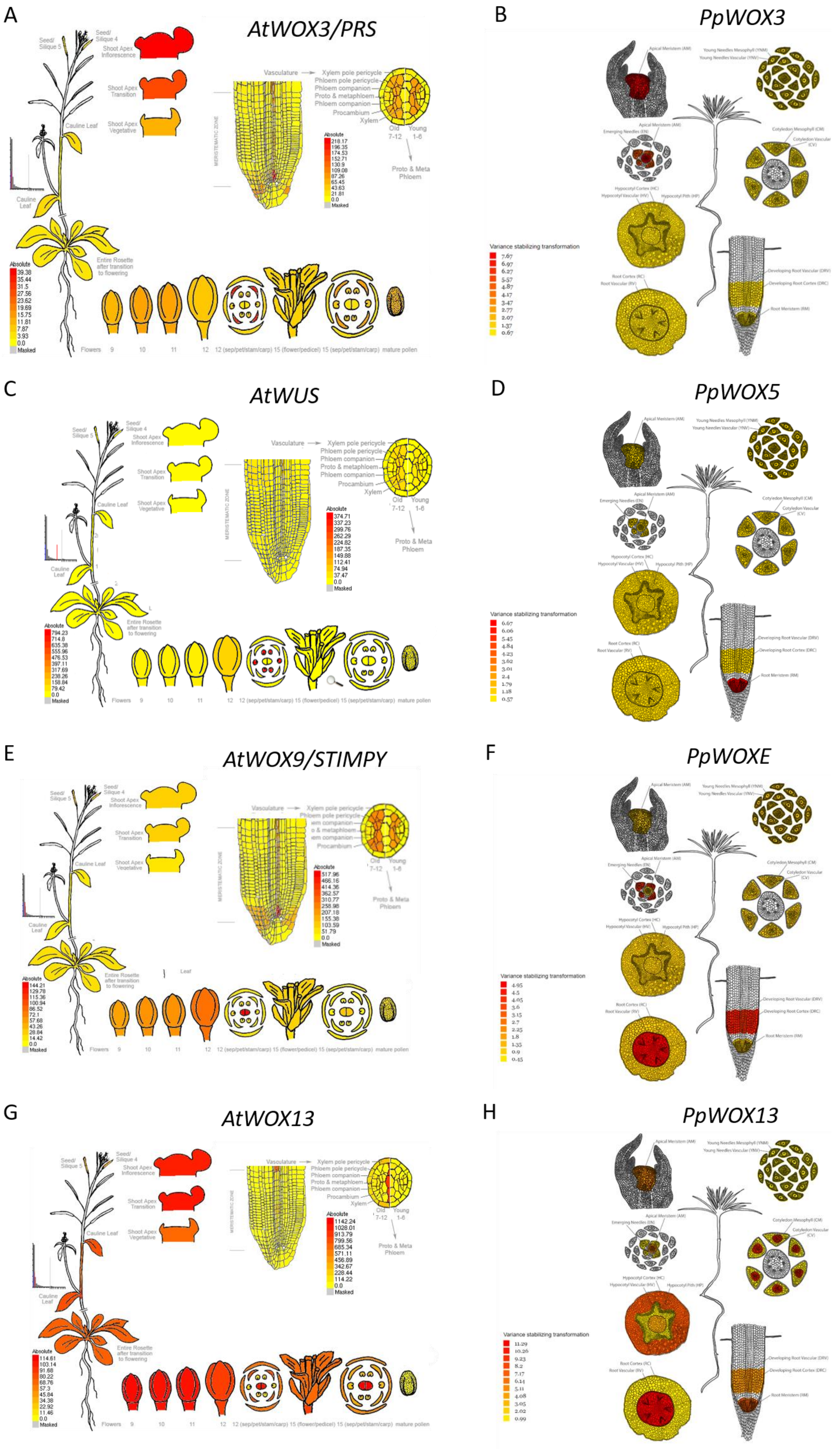

Suppl. Figure 2

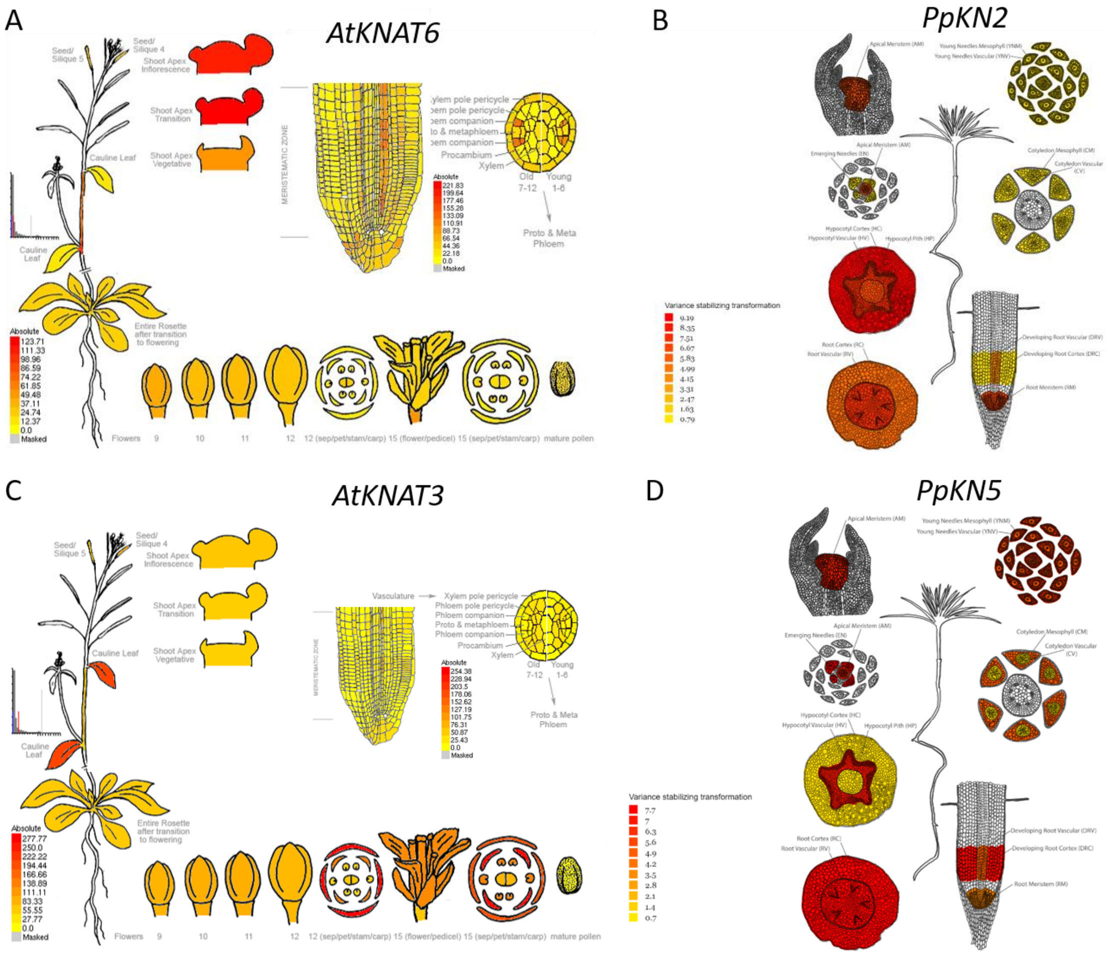

Supplement: Supplementary file 1 [file genes-12-00438-s001.pdf]
